# Mapping of Two Major QTLs Controlling Flowering Time in *Brassica napus* Using a High-Density Genetic Map

**DOI:** 10.3390/plants11192635

**Published:** 2022-10-07

**Authors:** Lei Chen, Weixia Lei, Wangfei He, Yifan Wang, Jie Tian, Jihui Gong, Bing Hao, Xinxin Cheng, Yingjie Shu, Zhixiong Fan

**Affiliations:** 1 College of Agriculture, Anhui Science and Technology University, Fengyang 233100, China; 2 Crop Institute, Anhui Academy of Agricultural Sciences, Hefei 230031, China; 3 Bengbu Ludu Crop Residue Biotechnology Co., Ltd., Bengbu 233000, China

**Keywords:** rapeseed, flowering time, quantitative trait locus, mapping

## Abstract

Research on the flowering habit of rapeseed is important for the selection of varieties adapted to specific ecological environments. Here, quantitative trait loci (QTL) for the days-to-flowering trait were identified using a doubled haploid population of 178 lines derived from a cross between the winter type SGDH284 and the semi-winter type 158A. A linkage map encompassing 3268.01 cM was constructed using 2777 bin markers obtained from next-generation sequencing. The preliminary mapping results revealed 56 QTLs for the days to flowering in the six replicates in the three environments. Twelve consensus QTLs were identified by a QTL meta-analysis, two of which (*cqDTF-C02* and *cqDTF-C06*) were designated as major QTLs. Based on the micro-collinearity of the target regions between *B. napus* and *Arabidopsis*, four genes possibly related to flowering time were identified in the *cqDTF-C02* interval, and only one gene possibly related to flowering time was identified in the *cqDTF-C06* interval. A tightly linked insertion–deletion marker for the *cqFT-C02* locus was developed. These findings will aid the breeding of early maturing *B. napus* varieties.

## 1. Introduction

Flowering is an important growth stage of higher plants, as it coincides with the transition from vegetative growth to reproductive growth. This transformation process determines the timing of flowering, which is important for ensuring high yields and the quality of crops [1]. Several studies of the model plant *Arabidopsis* have been conducted to clarify the genetic architecture of flowering time regulation, and these studies have resulted in the identification of at least 300 genes involved in flowering initiation. Environmental plasticity in this trait was also described [2], and the analysis of several *Arabidopsis* flowering time mutants by Koornneef et al. (2004) greatly contributed to our understanding of flowering initiation [3]. Six flowering time pathways have been proposed based on plant genetics and molecular biology studies in the model species *Arabidopsis thaliana*: the vernalization pathway, the photoperiod pathway, the gibberellin pathway, the autonomous pathway, the ambient temperature pathway, and the endogenous pathway [4,5]. This research on *Arabidopsis* has provided a solid foundation for dissecting the genetic and molecular bases of flowering time control in crops, especially for Brassicaceae crops, which show a high degree of evolutionary conservation in their molecular pathways with *Arabidopsis* [6].

Rapeseed (*Brassica napus* L.) is an important member of the family Brassicaceae and a major source of high-quality vegetable oil and protein-rich animal feed worldwide [7]. The allotetraploid *B. napus* was formed on the Mediterranean coast through a few independent natural hybridization events between the ancestral diploids *Brassica rapa* and *Brassica oleracea*, as well as chromosome doubling approximately 7500 years ago [8]. Natural variation in flowering time has promoted the ecological adaptability of rapeseed and facilitated its spread to subtropical and temperate regions. Vernalization is one of the many factors affecting flowering time in *B. napus*. Rapeseed germplasm can be divided into three ecotypes according to the relative importance of a vernalization response for normal flowering: the spring type, semi-winter type, and winter type [9,10,11]. Spring-type accessions flower normally without vernalization treatment, semi-winter accessions require moderate vernalization to promote flowering, and winter-type accessions depend highly on prolonged low temperatures for flowering [10,11]. Even within ecotypes, flowering time can vary to mediate adaptation to different climates or farming systems. Therefore, achieving an optimal flowering time is an important objective for rapeseed breeders.

Several flowering time genes and loci have been identified in *B. napus* over the past decades. There are two strategies that can be used to clone flowering time genes. The first strategy is homology cloning, which is based on the conservation of flowering time genes between *Arabidopsis* and *Brassica*. Several *FLC* (*FLOWERING LOCUS C*) paralogs in *B. napus* (*BnFLC1-5*) have been characterized by complementation tests in transgenic *Arabidopsis* using this approach [12]. Zou et al. (2012) also used this strategy to clone nine *FLC* homologs from the rapeseed genome and map them to six of 19 chromosomes [13]. Yin et al. (2021) cloned *BnaFLC.A10* and *BnaFLC.A2* in spring, winter, and semi-winter rapeseed and found that these two genes are required for the establishment of winter rapeseed crop phenotypes [14]. The second strategy involves using different populations to locate quantitative trait loci (QTLs) at the flowering stage in *B. napus* via forward genetic approaches. Parental linkage mapping analysis has been conducted in many of these studies. Long et al. (2007) showed that a major flowering time QTL, *qFT10-4*, which explained more than 50% of the phenotypic variation in flowering time during the spring environment, was located on chromosome A10 in the TN double haploid (DH) population [15]. Wang et al. (2009) identified six *BnFT* paralogs from the genome of *B. napus*, and three of them were associated with two major QTL clusters for flowering time [16]. Raman et al. (2013) constructed a DH population and found at least 20 QTLs in this population by QTL testing at the flowering stage [17]. Chen et al. (2018) successfully cloned the flowering time genes *BnFLC.A2* and *BnFLC.C2* using two pairs of near-isogenic lines [18]. Tudor et al. (2020) used the F_2_ population and QTL-seq to locate two flowering genes, *BnaFLC.A02* and *BnaFT.A02*, in the 10-Mb interval of the A02 chromosome [19]. Song et al. (2021) used the DH line to locate two major QTL sites for flowering time and identified four flowering-related candidate genes using transcriptome analysis, including *EMF1*, *NF-YA1*, *HAP2B*, and *COL9* [20]. Xu et al. (2020) discovered a new QTL for flowering time on chromosome A09 using F_2_ and its F_2:3_ line [21]. Fang et al. (2022) used the DH line to analyze lines with different combinations of the three flowering genes *BnFLC.A2*, *BnFLC.C2*, and *BnFLC.A3b* and concluded that the potential heterosis of flowering genes can regulate the development of *B. napus*, early flowering (maturity), and high yield, which can facilitate the early flowering of crops [22]. Association mapping analyses of natural populations have been conducted in recent years. Many single-nucleotide polymorphism (SNP) loci have been found to be associated with flowering time genes, such as *BnaFRI.A03*, *BnVIN3-C03*, *BnaFLC.A02*, *BnaFLC.A03*, *BnaFLC.C02*, and *BnaFLC.A10* et al. These SNP markers are significantly related to the flowering period and can be used for the whole-genome selection breeding of *B. napus* [23,24,25,26,27,28,29]. For example, Xu et al. (2016) conducted a genome-wide association study (GWAS) analysis and identified 41 SNPs associated with flowering time. Three SNPs were located in the *BnFLC.A10* region. Overall, these findings suggest that multiple copies of *FLC* homologs in *B. napus*, *B. rapa*, and *B. oleracea* have retained the function of *FLC* [24]. Helal et al. (2021) detected 15 and 37 QTLs from SNP and haplotype-based GWAS analyses, respectively, including the newly discovered loci *FT.A07.1*, *FT.A08*, *FT.C06*, and *FT.C07*, which regulate flowering time [27].

In this study, we used a high-density genetic linkage map to identify two major flowering time QTLs on chromosomes C02 (*cqDTF-C02*) and C06 (*cqDTF-C06*), which are two environmentally stable QTLs. We also predicted candidate genes for these QTLs and developed a closely linked insertion–deletion (InDel) marker for the *cqDTF-C02* locus. The results of this study enhance our understanding of the molecular basis for the regulation of flowering time and provide molecular markers that could be useful for the breeding of flowering time traits in rapeseed.

## 2. Results

### 2.1. Phenotyping of Two Parents and the 158A-SGDH Population 

**Figure 1:**
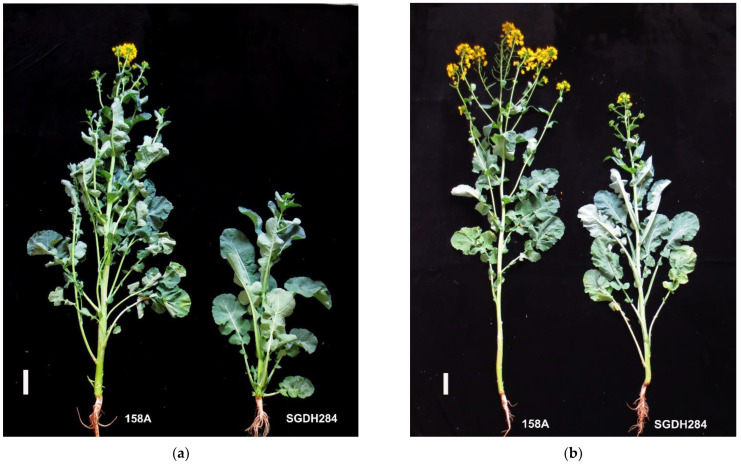
Phenotype of early-flowering parent 158A and late-flowering parent SGDH284 of Brassica napus in 2020. (**a**) A phenotypic picture taken on 152 days after sowing in a semi-winter rape environment. At this time, the early flowering parent 158A had already bloomed. (**b**) A phenotypic picture of a semi-winter rapeseed environment at 159 days after sowing, when the late-flowering parent SGDH284 began to bloom. Ruler = 10 cm.

The DH lines 158A and SGDH284 were used in this study (Figure 1a,b) in the semi-winter rape environment (Fengyang, Anhui). The difference in flowering time between these two materials was approximately 7–13 days, and this difference was significant (Table 1). We crossed the early flowering 158A (female parent) and the late flowering SGDH284 (male parent) to obtain the F_1_ generation. The anthers of the F_1_ generation were collected for microspore culture and doubled with colchicine to obtain the DH population, which was named 158A-SGDH in this study. Days-to-flowering (DTF) phenotypic data were collected from the 178 DH lines in three experiments conducted over three years (Table 1 and Table S1). 

**Figure 2:**
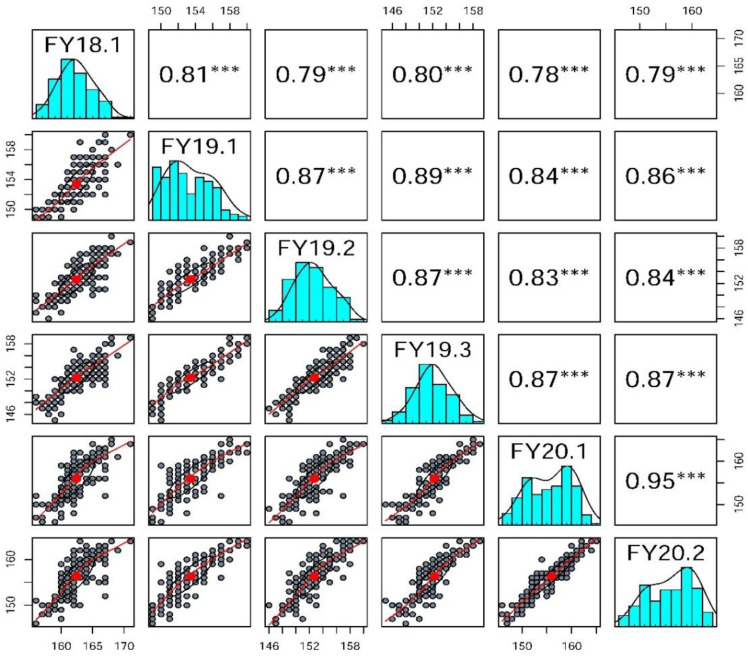
Correlation analyses between days-to-flowering phenotypes of the 158A-SGDH population. Among them, it was repeated once in 2018–2019, three times in 2019–2020, and two times in 2020–2021. *** indicates *p* < 0.001.

The flowering phenotype of the 158A-SGDH population showed a normal distribution and ranged from 145 to 171 days for the six replicates over three years, indicating that the DTF phenotype is a quantitative trait controlled by multiple genes (Figure 2 and Figure S1). Moreover, the DTF of the 158A-SGDH population for the period of 2019–2020 was significantly earlier than that for the period of 2018–2019, which might have stemmed from the warmer ambient temperature in the winter of 2019 (Figure S1). There was a significant positive correlation in the DTF phenotype among the six replicates in the three environments (r^2^ = 0.78–0.95, *p* < 0.001) (Figure 2). The broad-sense heritability of DTF was 91.52%.

### 2.2. High-Density Genetic Linkage Map Construction

To construct a high-density linkage map, next-generation sequencing (NGS) was performed on a total of 178 individuals from the DH population, and a total of 360.87 Gb clean data were generated (Table S2). The average quality score of Q20 was over 98.69%, the GC content was approximately 39.09% (Table S2), and the average sequencing depth was 1.70× for every individual (Table S2). For the parents 158A and SGDH284, 5,064,588,459 bp and 5,362,611,753 bp of clean data were detected, and the sequencing depths were 3.67× and 4.19×, respectively (Table S2).

**Figure 3:**
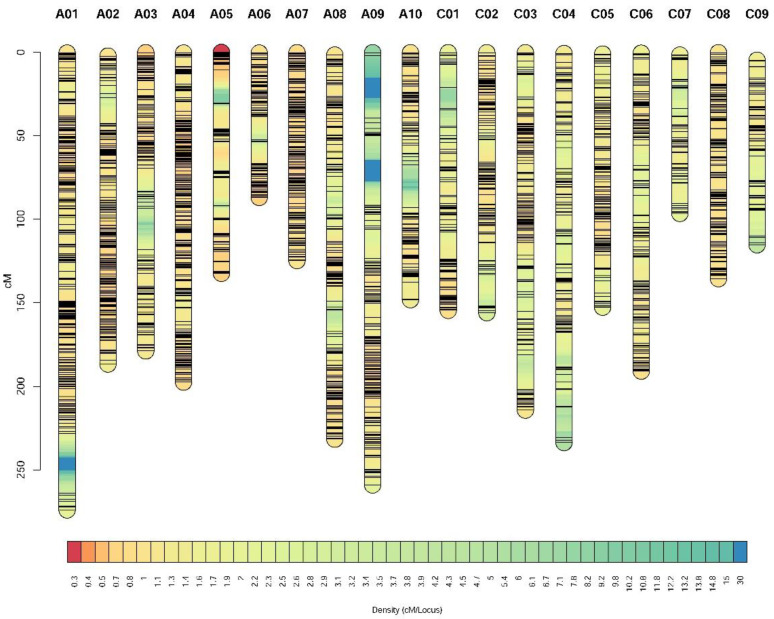
Genetic linkage map of the 158A-SGDH population. The name of each linkage group is shown above it. The ruler on the left indicates the genetic position in centimorgans (cM). The color scale at the bottom represents the density of markers (cM/locus).

According to the NGS data of the two parental lines and 178 individuals, a total of 8,780,700 SNPs were identified, and 946,690 SNPs were used for map construction. A total of 13,395 bin markers were obtained based on an analysis of every 15 SNP, each of which was considered a bin [30], and these were subsequently used for genotyping in 178 individuals. Finally, a total of 2777 bin markers were anchored to 19 linkage groups (Table S3). The total length of the linkage groups was 3268.01 cM, and the average distance between the markers was 1.18 cM (Figure 3, Tables S3 and S4). Of the 19 linkage groups, the longest was A01 (273.90 cM), followed by A09 (259.02 cM), and the shortest was A06 (86.89 cM). The linkage group with the largest number of bin markers was A01 (254 bins), followed by A04 (243 bins), and linkage group C07 had the lowest number of bins (56 bins) (Tables S3 and S4). 

To assess the quality of the genetic map, we analyzed the correlation between the bin markers in the genetic map and their positions on the physical map (Figure S2, Tables S5 and S6). The mean value of the Pearson correlation coefficients between the genetic map and the physical map was 93.8%, indicating high collinearity between them (Figure S2 and Table S7). The above results indicate the robustness and accuracy of the constructed genetic map.

### 2.3. QTL Mapping for DTF

**Figure 4:**
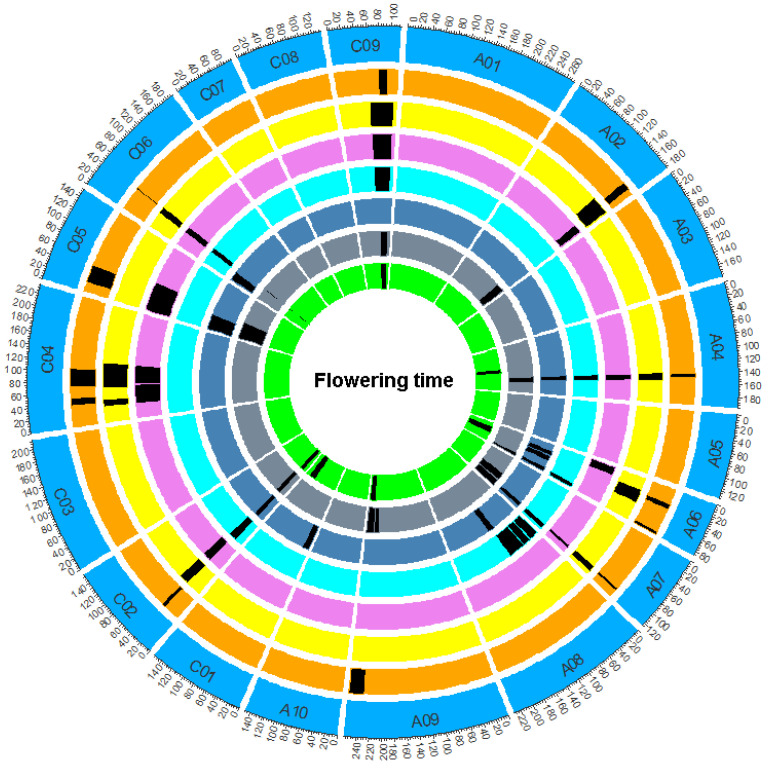
Schematic diagram of QTL mapping for flowering time in the 158A-SGDH population. The circles from outside to inside show the consensus QTL and the identified QTL of the flowering time. The outermost circle is the chain group, the second orange circle represents the consensus QIL position, and the third circle inward represents different repetitions in different environments, followed by FY20.1, FY20.2, FY19.1, FY19.2, FY19.3, and FY18.1. The black area in the circle indicates the positions of the consensus QTL and the identified QTL.

WinQTLCart 2.5 software’s standard model of composite interval mapping (model 6) was used for QTL analysis. The phenotypic data at the flowering stage were the phenotypic values of 178 lines with six replicates in three years. A total of 56 QTLs at the flowering stage were detected by QTL analysis (Figure 4, Table S8). The proportion of phenotypic variation explained by a single QTL ranged from 2.70% to 32.04%. The identified QTLs were distributed over 12 linkage groups, and the confidence intervals (CIs) of the QTLs in the different experiments overlapped. Among the 56 QTLs, 44 could be integrated by meta-analysis into 12 reproducible consensus QTLs named *cqDTF-A02*, *cqDTF-A04*, *cqDTF-A06-1*, *cqDTF-A06-2*, *cqDTF-A07*, *cqDTF-A09*, *cqDTF-C02*, *cqDTF-C04-1*, *cqDTF-C04-2*, *cqDTF-C05*, *cqDTF-C06*, and *cqDTF-C09* (Table 2). Among these shared QTLs, *cqDTF-C02* was detected in all six replicates, and *cqDTF-A04*, *cqDTF-A07*, *cqDTF-C06*, and *cqDTF-C09* were detected in all five replicates. Four QTLs (*cqDTF-A02*, *cqDTF-A06-1*, *cqDTF-A06-2*, and *cqDTF-C05*) were detected in three replicates, and *cqDTF-A09*, *cqDTF-C04-1*, and *cqDTF-C04-2* were detected in two replicates (Table 2, Table S8). 

**Figure 5:**
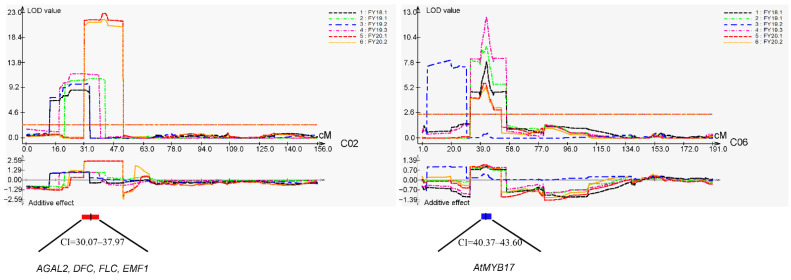
Two major QTL sites for flowering time, C02 and C06. The upper curve represents the QTLs identified in six replicates in the three environments, and the lower curve represents the additive effect of the QTLs with the same color. The major QTLs are shown under the confidence intervals. Candidate genes related to flowering time are shown under the major QTL.

Two stable major QTLs at the flowering stage (*cqDTF-C02* and *cqDTF-C06*) were detected. The *cqDTF-C02* locus explained 13.54–32.04% of the phenotypic variation, and *cqDTF-C06* explained 6.07–16.13% of the phenotypic variation (Figure 5, Table 2). The results of the meta-analysis show that the peak value of the *cqDTF-C02* locus was in the genetic linkage map at 34.02 cM, the CI was 30.07–37.97 cM, and the two sides were labeled chrC02_bin6298 and chrC02_bin6308; the interval of the locus was from 1,885,589 to 2,918,506 bp (Figure 5, Table S6). *cqDTF-C06* was another major QTL, with a peak value at 41.98 cM in the genetic linkage map; the CI was 40.37–43.60 cM, the two sides were labeled chrC06_bin10767 and chrC06_bin10772, and the corresponding segment was within the physical interval of 33,585,972–34,116,003 bp (Figure 5, Table S6).

### 2.4. Candidate Gene Prediction of the Two Major Flowering time QTL Regions

According to the *B. napus* ZS11 reference genome (http://yanglab.hzau.edu.cn/BnIR/ accessed on 10 March 2022), 195 genes were predicted in the 1.03-Mb *cqDTF-C02* region. Based on the micro-collinearity of the target region between *B. napus* and *Arabidopsis*, we detected four genes that might be associated with flowering time: *BnaC02G0032100ZS* (*AT5G08370*/*AGAL2*), *BnaC02G0038900ZS* (*AT5G10130*/*DFC*), *BnaC02G0039100ZS* (*AT5G10140*/*FLC*), and *BnaC02G0046300ZS* (*AT5G11530*/*EMF1*) (Figure 5, Table S9). In addition to the above four genes that might be related to flowering time, 29 genes with unknown functions were identified in this segment (Table S9). Ke et al. (2020) sequenced the transcriptomes of *B. napus* 158A and SGDH284 [31]. The analysis of the transcriptome data of parental 158A and SGDH284 at the seedling stage revealed four flowering-related genes in this QTL region (*BnaC02G0032100ZS*, *BnaC02G0038900ZS*, *BnaC02G0039100ZS*, and *BnaC02G0046300ZS*). The fragments per kilobase transcript per million reads (FPKM) values of the *BnaC02G0032100ZS* gene were 71.96 and 74.88 in 158A and SGDH284, respectively. The FPKM values of *BnaC02G0038900ZS*, *BnaC02G0039100ZS*, and *BnaC02G0046300ZS* were all less than 5 in the 158A parent; however, the FPKM values of these genes were higher in the SGDH284 parent (20.64, 277.51, and 31.31, respectively) (Table S11).

According to the ZS11 reference genome, 65 genes were identified in the 530-kb interval in the *cqDTF-C06* region. We found that one of these genes might be related to flowering time, namely *BnaC06G0229100ZS* (*AT3G61250*/*AtMYB17*) (Figure 5, Table S10). The FPKM value of *BnaC06G0229100ZS* was less than 5 in both the 158A and SGDH284 parents (Table S11). In addition, 15 genes with unknown functions were identified in this QTL region (Table S10).

### 2.5. InDel Markers Linked to Flowering Time in the Target Intervals

**Figure 6:**
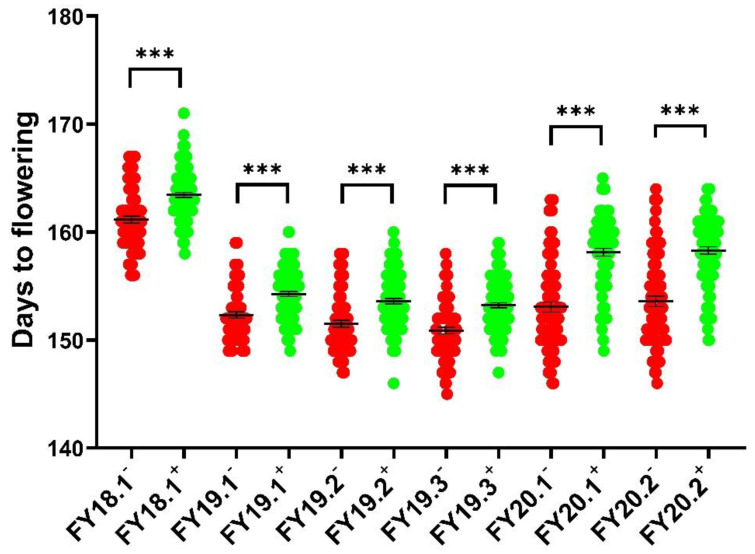
Six replicates of the 158A-SGDH population were analyzed for days to flowering using the InDel marker C2-5 at the *cqDTF-C02* locus. − indicates individual plants without *cqDTF-C02* locus, + indicates individual plants with *cqDTF-C02* locus. Days to flowering were measured as mean ±SEM *** indicates *p* < 0.001.

InDel markers for *cqDTF-C02* and *cqDTF-C06* were developed based on the Illumina sequencing data of 158A and SGDH284 using the ZS11 reference genome. We developed nine InDel markers for the *cqDTF-C02* locus, and one of the primers with parental polymorphism and a strong band pattern was C2-5. We analyzed a small population of 10 extremely early and 10 extremely late flowering plants with the C2-5 marker and found that the marker was associated with flowering time (Figure S3). The flowering times of six replicates of the 158A-SGDH population were studied using the InDel marker C2-5 at the *cqDTF-C02* locus. The flowering times of the lines carrying this locus and those without this locus significantly differed (Figure 6). Primers that were polymorphic between the parents could not be developed for the *cqDTF-C06* locus.

## 3. Discussion

### 3.1. Importance of Flowering Time in the Crops

An important goal of crop breeding is to improve crops to better adapt to the local environment and climate, so as to breed new varieties [32]. Previous studies have shown that early flowering and early maturity of *B. napus* had a significant positive correlation, and early maturity of *B. napus* could be bred through the selection of early flowering materials [33,34]. Flowering time genes have been reported to regulate crop yield in rice, tomato, and rapeseed [22,35,36,37,38,39]. Therefore, the identification of new flowering regulatory loci can help to elucidate the genetic basis of flowering and then select varieties adapted to different geographic regions. In this study, we identified two main QTLs for flowering time, and found that the trait had a high heritability (91.52%) based on three years of six replicates of flowering data, which is consistent with the results of the study by Li et al. (2018) and Helal et al. (2021) [11,27]. The high heritability at the flowering time will help us to carry out marker-assisted selection breeding for this trait.

### 3.2. Construction of the High-Density Genetic Map for the 158A-SGDH Population

QTL mapping is an effective method for analyzing complex quantitative traits. The construction of high-density genetic linkage maps can increase the accuracy of QTL mapping. In recent years, many rapeseed studies have successfully mapped flowering time QTLs using high-density maps [20,21,23,24,25,26,27,28,29,40]. In this study, after constructing a DH population with 178 lines, we constructed a high-density genetic linkage map of this DH population using NGS technology. The map was 3268.01 cM in length and contained 2777 bin markers with an average marker interval of 1.18 cM (Figure 3 and Table S3). However, some linkage groups did not completely cover the physical map, such as A03, A07, A09, C02, C03, C04, C06, and C08 (Figure S2). This might be explained by the low individual sequencing depth of the DH population; the average sequencing depth was 1.70× for each individual (Table S3). To increase the accuracy of the analysis, the sequencing depths for the 158A and SGDH284 parents were 3.67× and 4.19×, respectively (Table S2).

### 3.3. Analysis of Two Major Flowering Time QTLs in the 158A-SGDH Population

A total of 56 QTLs for flowering time were detected by QTL analysis. Two major QTLs, *cqDTF-C02* and *cqDTF-C06*, were identified by meta-analysis (Table 2); these QTLs could explain 13.54–32.04% and 6.07–16.03% of the phenotypic variation, respectively (Figure 5, Table 2). These two major QTLs were detected in six and five replicates, respectively, and they were also detected stably and in the same direction, indicating that the two QTLs changed little among years; this facilitated the fine mapping of QTLs and gene cloning (Figure 5, Table 2). The *cqDTF-C02* locus was detected in different populations, indicating that this QTL shows extensive allelic variation among different varieties [17,18,23]. Raman et al. (2016) conducted a GWAS analysis and found that *FLC2* (*Bna.FLC.A02*/*C02*) was responsible for 22% of the variation in flowering time under non-vernalized conditions in a diverse population of spring-type rapeseed [23]. Chen et al. (2018) used a pair of near-isogenic materials to map a major QTL in the C02 linkage group, which could explain 47.9% of the phenotypic variation [18]. They experimentally showed that *BnFLC.C2* was the target gene in this locus. Another major QTL, *cqDTF-C06*, detected in five replicates in the three years of this study but not in previous studies, was identified as a novel QTL for flowering time. *cqDTF-C06* is likely derived from a novel mutation, and more natural germplasm should be examined to determine whether it represents a rare mutation and can be exploited for the breeding of early maturing rapeseed.

These findings indicate that our experimental results are reliable. However, there were no overlapping loci with *cqDTF-C06*, suggesting that this might be a new QTL for flowering time. Thus, our findings have increased our knowledge of the QTLs for flowering time in rapeseed.

### 3.4. Analysis of Candidate Genes Associated with the Two Major Flowering Time QTLs

In *Arabidopsis*, the molecular network of flowering time was summarized by Srikanth and Schmid (2011) [4]. Cultivated Brassica species, such as *B. rapa*, *B. oleracea*, and *B. napus*, all belong to the family Brassicaceae [41]. Because Brassica crops are closely related to the model plant *Arabidopsis*, many studies have analyzed the flowering genes of *B. napus* and compared them with those in *Arabidopsis*. In our study, we mapped two major flowering time QTLs, *cqDTF-C02* and *cqDTF-C06*, in rapeseed using a QTL mapping strategy. 

In the *cqDTF-C02* region, we identified four genes involved in flowering: *AT5G08370*/*AGAL2*, *AT5G10130*/*DFC*, *AT5G10140*/*FLC*, and *AT5G11530*/*EMF1*. The corresponding genes in rapeseed are *BnaC02G0032100ZS*, *BnaC02G0038900ZS*, *BnaC02G0039100ZS*, and *BnaC02G0046300ZS* (Figure 5, Table S9). The above four candidate genes in rapeseed are homologous to the genes that inhibit flowering in *Arabidopsis*, including *AT5G10140*/*FLC* and *AT5G11530*/*EMF1*. The genes that promote flowering are *AT5G08370*/*AGAL2* and *AT5G10130*/*DFC*. The *AT5G08370*/*AGAL2* gene is a member of the glycoside hydrolase family 27 (GH27) and functions as an α-galactosidase. Mutations of this gene delay flowering by up to 3 weeks, and the total number of leaves in the rosette was 17–21 in mutant plants compared to 10–13 in wild-type plants [42]. *AT5G10130*/*DFC* is located downstream of the *FLC* gene, which Swiezewski et al. (2007) found could lead to reductions in *FLC* mRNA and early flowering in *Arabidopsis* [43]. *AT5G10140*/*FLC* (*FLOWERING LOCUS C*) is a key regulator of flowering time in the vernalization pathway; *FLC* encodes a MADS-box transcription factor that delays flowering by blocking the transcription of *FT*, *SOC1*, and *FD* [44,45,46]. *AT5G11530*/*EMF1* is a subunit of a polycomb repressive complex, which is involved in the repression of *FT* expression, and thus, prevents photoperiod-independent flowering. *EMF1* also regulates *AG*, *AP3*, *PI*, *SPL*, and *MIR172* expression [47]. 

In previous studies, *FLC* has been identified as the key gene involved in the vernalization pathway of the *Arabidopsis* flowering regulatory network; it has thus been well studied. The *FLC* copy located on C02 has been mapped to several QTLs [17,18,23], including the main QTL *cqDTF-C02* in this study. Candidate genes with high homology to the flowering time gene *AT5G10140* (*FLC*) of *Arabidopsis* were also detected, including *BnaC02G0039100ZS*. Transcriptome data analysis of the 158A and SGDH284 parents at the seedling stage showed that the expression of *BnaC02G0039100ZS* differed in the parents. The FPKM value was less than 5 in the 158A parent, and the FPKM value in the SGDH284 parent was 277.51. *FLC* homologous genes on A2 and C2 have been shown to alter the flowering time of rape [18,23]. In *Arabidopsis*, *FLC* directly binds to the promoter region of *SOC1* and to the first intron of *FT*, which prevents the expression of these genes [38,48]. The repression of *SOC1* and *FT* expression delays the expression of the floral meristem identity genes *LFY* and *AP1*, which increases the length of the vegetative phase and thus delays flowering [49,50,51]. Therefore, *BnaC02G0039100ZS*, which is homologous to *AT5G10140* (*FLC*), might be the target gene of the *cqDTF-C02* region. 

In the *cqDTF-C06* region, only one gene related to flowering, *AT3G61250*/*AtMYB17*, was observed, and the corresponding gene in rapeseed is *BnaC06G0229100ZS* (Figure 5, Table S10). *AT3G61250*/*AtMYB17* is a target of the meristem identity regulator *LEAFY* (*LFY*); it plays a role in the meristem identity transition from vegetative growth to flowering [52,53]. 

Flowering-related genes might also be found in the *cqDTF-C02* and *cqDTF-C06* QTL segments, as they contained 29 and 15 unknown genes, respectively (Tables S9 and S10). We plan to construct near-isogenic lines to further narrow the candidate interval through fine mapping. Additional work is needed to functionally verify possible candidate genes; genetic complementation experiments also need to be conducted.

## 4. Materials and Methods

### 4.1. Plant Materials

A DH population consisting of 178 lines was derived from a cross between a male SGDH284 parent and a female 158A parent; these two DH lines were selected from a microspore culture of winter rapeseed Sollux and semi-winter rapeseed zhongyou9988, respectively. The Sollux was provided by Dr. Xiyuan Ni (Zhejiang Academy of Agricultural Sciences), and the zhongyou9988 was a commercial variety bred by the Oil Crops Research Institute of the Chinese Academy of Agricultural Sciences [31].

### 4.2. Field Experiment and Phenotypic Measurements

The 178 DH lines, together with their parental lines SGDH284 and 158A, were grown under natural field conditions at the Experimental Station of Anhui Science and Technology University, Fengyang, China. All the lines were arranged in a completely randomized block design with one replicate in 2018-2019, three replicates in 2019–2020, and two replicates in 2020–2021. Each line was planted in two rows, with 10-12 plants in each row. The length and width of the rows were 2 m and 0.3 m, respectively. Field management followed standard agricultural practices. Three natural environments with six replicates, FY18.1 (Fengyang, 2018–2019), FY19.1/19.2/19.3 (Fengyang, 2019–2020), and FY20.1/20.2 (Fengyang, 2020–2021), were used to study the DTF trait. The DTF of the parents and the DH population were recorded as the number of days from the day of sowing to the day when 50% of the rapeseed plants exhibited at least one open flower in a plot [10]. The broad-sense heritability was estimated using linear mixed models in the lme4 package [54].

### 4.3. Genome Sequencing and Genotyping

Genomic DNA from the parents of the 178 DH lines was extracted using a modified cetyltrimethyl ammonium bromide method [55] and stored at −20 °C. The sequencing was conducted by Personal Biotechnology Co., Ltd. Shanghai, China. Using NGS, a library of 400 inserts was constructed for the parents and the 178 DH lines. Paired-end sequencing of these libraries was then conducted using the Illumina NovaSeq sequencing platform. The BWA-MEM (0.7.12-r1039) [56] program was then used to compare the filtered high-quality data against the reference genome (the *B. napus* ZS11 reference genomes [57,58]) using default parameters. Picard 1.107 software was used to sort the SAM files and convert them into BAM files. Reads near InDels are most prone to mapping errors. To minimize the identification of SNPs caused by mapping errors, the reads near InDels were re-compared to improve the accuracy of the SNP calling. The Indel realigner command in the GATK program was used to re-compare all reads near InDels to improve the accuracy of SNP prediction. After genome sequencing, a total of 8,780,700 SNPs were obtained through analysis and screening. To construct a genetic map, the 8,780,700 SNPs obtained were filtered by retaining ones in which (1) the sites are homozygous in the two parents and inconsistent between the parents; (2) sites where the offspring’s sequencing depth is greater than 2; and (3) sites where the offspring’s deletion rate is less than 0.5. After filtering, a total of 946,690 SNPs were obtained. All 15 SNPs were considered a bin, and 15 SNPs were used as a sliding window from the beginning to the end of the linkage group [30]. Finally, 13,395 bin markers were obtained. Fifteen SNPs corresponded to 30 alleles, and these 30 loci were defined as 0 if the genotype of parent 1 was greater than or equal to 24, defined as 2 if the genotype of parent 2 was greater than or equal to 24, and 1 if it was between the two [30]. In the QTL mapping, genotype 1 was treated as a deletion type. Genotypes were converted into the input file format of MSTmap software. 

### 4.4. Linkage Map Construction and QTL Mapping

The software MSTmap was used to classify and map linkage groups [59]. The parameters were set as follows: the cut-off p-value was 1E−6, the no-map-dist was 30.0, and the no-map-size was 2. The values of the recombination fractions were converted into genetic map distances (cM) via the Kosambi mapping function [60]. 

QTL analyses for the DTF trait were conducted by composite interval mapping [61] using WinQTL cartographer 2.5 software (http://statgen.ncsu.edu/qtlcart/WQTLCart.htm/ accessed on 18 May 2021). The walking speed was set to 1 cM. The LOD threshold for the DTF trait was determined by permutation analysis with 1000 repetitions. Significant QTLs repeatedly identified in different environments were integrated into consensus QTLs by a QTL meta-analysis using BioMercator 4.2 [62,63]. These consensus QTLs were divided into two groups: major QTLs consisting of R^2^ > 20% or R^2^ > 10% in two or more environments, and the rest of the QTLs, which were designated as minor [64].

### 4.5. Candidate Gene Analysis within Target Intervals

All the sequences of the annotated genes from the candidate regions were downloaded from the *B. napus* reference genome, ZS11 (http://yanglab.hzau.edu.cn/BnIR and http://cbi.hzau.edu.cn/bnapus/index.php/ accessed on 10 March 2022) [65,66], and aligned against the genome sequence of *Arabidopsis* by local BLAST analysis. 

The genomic sequences of the predicted genes were submitted to NCBI (http://www.ncbi.nlm.nih.gov/ accessed on 12 March 2022) and TAIR (www.arabidopsis.org/ accessed on 12 March 2022) for homolog search and basic functional analysis. Genes from *B. napus* with the highest sequence similarity to genes in *Arabidopsis* were designated as orthologous genes. *Arabidopsis* genes associated with flowering time traits were retrieved. Finally, their *B. napus* homologs within the CIs of target QTLs were designated as putative candidate genes for the QTLs.

### 4.6. Development of InDel Markers in the Candidate Regions

InDel polymorphic sites between two parents in the vicinity of the major QTL region were selected and converted into PCR-based markers according to the re-sequence data. Polymorphism analysis between parents was performed using these markers. Markers with clear polymorphic bands between the parents were then used to analyze a small population of 10 extremely early flowering individuals, 10 extremely late flowering individuals, and both parents. For the *cqDTF-C02* site, one marker with better bands was developed and named C2-5. The sequence of the left primer was 5′-CGTGTCAAGTCTGCATTGTTGT-3′, and that of the right primer was 5′-TTCCTGCCTTATCCATCCCA-3′.

## 5. Conclusions

We obtained 12 reproducible consensus QTLs during the flowering stage of rapeseed through preliminary mapping and meta-analysis of the QTLs of the 158A-SGDH population, and there were two major consensus QTLs, *cqDTF-C02* and *cqDTF-C06*. The analysis of the two major QTL regions revealed five possible candidate genes in the two QTL intervals during the flowering stage. We also developed a molecular marker closely linked to *cqDTF-C02*, and this marker could be useful for marker-assisted selection. The results of this study will aid the cloning of flowering time-related genes of rapeseed, the development of functional markers of flowering time-related genes, and the breeding of early maturing rapeseed varieties. Molecular marker-assisted selection has the advantages of high accuracy, high efficiency, and low cost. This study provided excellent molecular markers for the improvement of early flowering and early maturity breeding of rapeseed, which could be directly applied to targeted marker-assisted breeding and the production of rapeseed.

## Figures and Tables

**Table 1:** Phenotypic variation of flowering time in the 158A-SGDH population and their parents.

Environment	Parents		158A-SGDH Lines			
	158A ^a^	SGDH284 ^a^	DH Lines Range	Mean ± SEM	Skewness	Kurtosis
FY18.1	159.33 ± 0.88	166.67 ± 0.67 ***	156–171	162.44 ± 0.21	0.14	−0.17
FY19.1	149 ± 0.00	157 ± 0.58 ***	149–160	153.44 ± 0.20	0.30	−0.77
FY19.2	149 ± 0.00	157 ± 0.58 ***	146–160	152.73 ± 0.21	0.18	−0.46
FY19.3	149 ± 0.00	157 ± 0.58 ***	145–159	152.25 ± 0.20	0.04	−0.04
FY20.1	149.33 ± 0.88	162.33 ± 0.33 ***	146–165	155.99 ± 0.33	−0.21	−0.97
FY20.2	149.33 ± 0.88	162.33 ± 0.33 ***	146–164	156.31 ± 0.33	−0.27	−0.92

The significance level by *t* test. *** *p* < 0.001. ^a^ Mean ± SEM, SEM means standard error of mean.

**Table 2:** Consensus QTLs for DTF identified by meta-analysis in the 158A-SGDH population.

QTL	Chr	Environment	CI (cM)	Peak	LOD	PVE (%) ^a^	Add ^b^
*cqDTF-A02*	A02	FY19.3/20.1/20.2	160.25–175.34	167.8	2.51–4.11	2.77–4.24	−0.56~−0.98
*cqDTF-A04*	A04	FY19.1/19.2/ 19.3/20.1/20.2	140.64–147.53	144.08	4.45–5.60	5.16–7.38	0.63~1.16
*cqDTF-A06-1*	A06	FY18.1/19.2/ 20.1	28.11–38.09	33.1	3.49–5.91	4.53–7.63	0.60~1.16
*cqDTF-A06-2*	A06	FY19.1/ 19.2/19.3	81.03–86.89	84.43	2.74–6.70	3.61–8.28	0.54~0.80
*cqDTF-A07*	A07	FY19.1/19.2/19.3/ 20.1/20.2	106.28–111.41	108.85	3.93–8.23	4.07–9.57	0.55~1.37
*cqDTF-A09*	A09	FY18.1/19.3	230.58–258.24	233.95	2.8–3.03	3.02–3.93	0.48~0.57
*cqDTF-C02*	C02	FY18.1/19.1/19.2/ 19.3/20.1/20.2	30.07–37.97	34.02	8.86–22.92	13.54–32.04	1.06~2.59
*cqDTF-C04-1*	C04	FY20.1/20.2	39.16–53.01	46.09	2.7–2.94	2.70–3.17	1.06~1.11
*cqDTF-C04-2*	C04	FY20.1/20.2	72.88–104.75	88.82	2.97–3.03	3.1–3.28	1.11~1.12
*cqDTF-C05*	C05	FY19.2/19.3//20.2	17.1–49.34	33.22	2.53–2.85	2.74–3.74	0.46~0.73
*cqDTF-C06*	C06	FY18.1/19.1/ 19.3/20.1/20.2	40.37–43.60	41.98	5.31–12.6	6.07–16.13	0.93~1.12
*cqDTF-C09*	C09	FY18.1/19.1/ 19.3/20.1/20.2	78.83–94.84	86.84	3.04–4.47	3.13–6.45	0.58~0.86

^a^ Proportion of the phenotypic variation explained by the QTL; ^b^ − indicate the direction of the additive effect.

## Data Availability

The data presented in this study are available in the article and Supplementary Materials. The BioProject accession number of the sequencing data of 180 materials of 158A-SGDH population and their parents is PRJNA885910 (a preview of the data can be viewed here: https://submit.ncbi.nlm.nih.gov/subs/bioproject/SUB12105022/overview/ accessed on 1 October 2022, https://www.ncbi.nlm.nih.gov/sra/PRJNA885910/ accessed on 1 October 2022).

## Supplementary Materials

The following supporting information can be downloaded at: https://www.mdpi.com/article/10.3390/plants11192635/s1, Table S1: Days to flowering of 178 individuals in the DH population in Fengyang in 2018-2019/2019-2020/2020-2021; Table S2: The sequence data quality of 178 individuals in the DH population and two parents; Table S3: Distribution of SNPs on 19 genetic linkage groups; Table S4: The bin marker information and the genotype of the 178 individuals in DH population and two parents; Table S5: The genetic position of all the mapped bin markers in each linkage group; Table S6: The physical position of all the bin markers in each chromosome; Table S7: The R^2^ value between the genetic map and physical map in each linkage group; Table S8: QTL detection of days to flowering in 158A-SGDH population; Table S9: The candidate gene information in the major *cqDTF-C02* QTL region; Table S10: The candidate gene information in the major *cqDTF-C06* QTL region; Table S11: The FPKM values of flowering time-related genes in two major QTL segments in parental 158A and SGDH284 seedling stage; Figure S1. Frequency distribution of phenotypes at flowering stage in Brassica napus 158A-SGDH population; Figure S2. Collinearity analysis of the genetic map and physical map in B. napus; Figure S3. A small population of 10 extremely early flowering and 10 extremely late flowering plants were analyzed by the *cqDTF-C02* locus-specific InDel marker C2-5. Remarks: Tables S1–S7 and Tables S9–S11 are in the Excel file named Supplementary Tables; Table S8 and Figures S1–S3 are in the Word file named Supplementary Information.

## Abbreviations

Flowering time (FT); genome-wide association study (GWAS); flowering time (FT); *FLOWERING LOCUS C* (*FLC*); *LEAFY* (*LFY*); quantitative trait locus (QTL); single nucleotide polymorphism (SNP); days to flowering (DTF); next-generation sequencing (NGS); fragments per kilobase transcript per million reads (FPKM).
