# Supplementary material for: Mapping of Two Major QTLs Controlling Flowering Time in Brassica napus Using a High-Density Genetic Map
Source: Plants (Basel). 2022 Oct 7;11(19):2635. doi: 10.3390/plants11192635 (PMC9571212; doi:10.3390/plants11192635)
Supplement: Supplementary file 1 [file plants-11-02635-s001.zip › Supplementary Figure1-3 TableS8.pdf]

Supplementary Materials

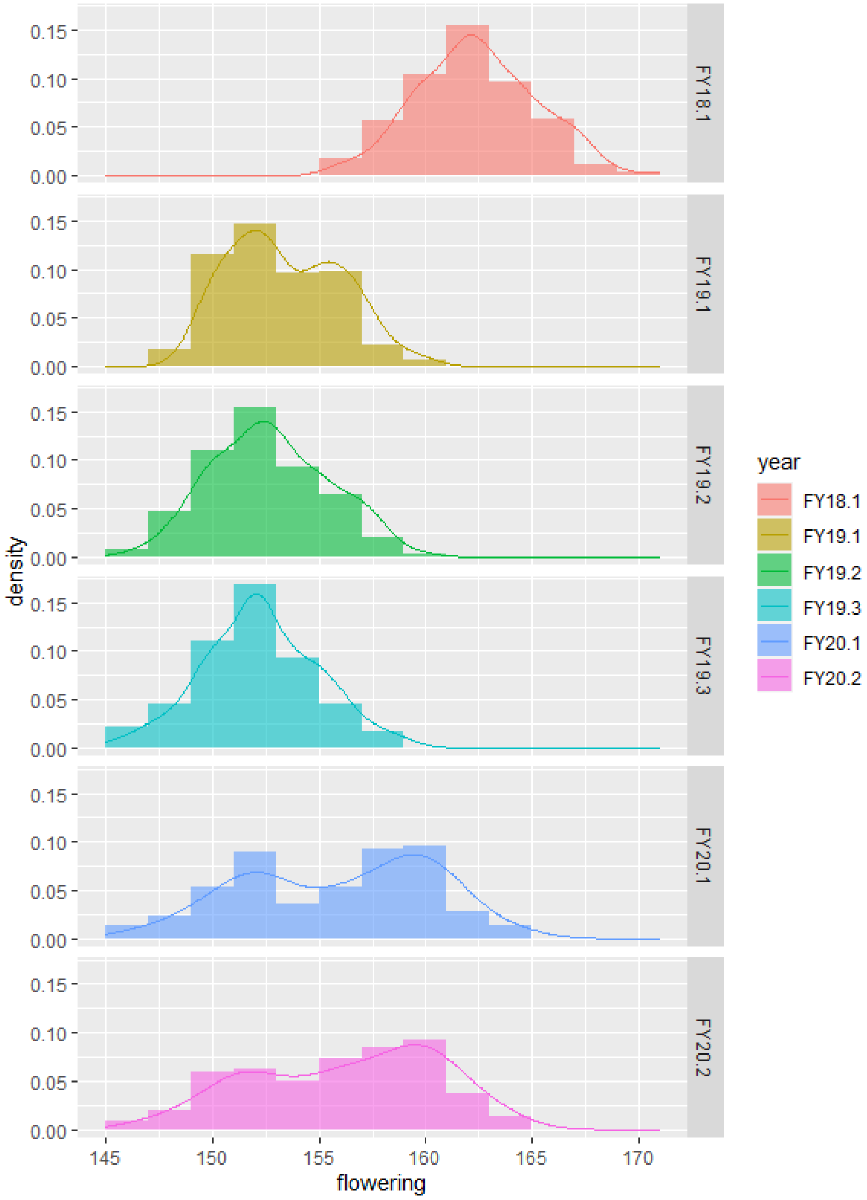

Figure S1. Frequency distribution of phenotypes at flowering stage in *Brassica napus* 158A-SGDH population with one replicate in 2018-2019, three replicates in 2019-2020, and two replicates in 2020-2021.

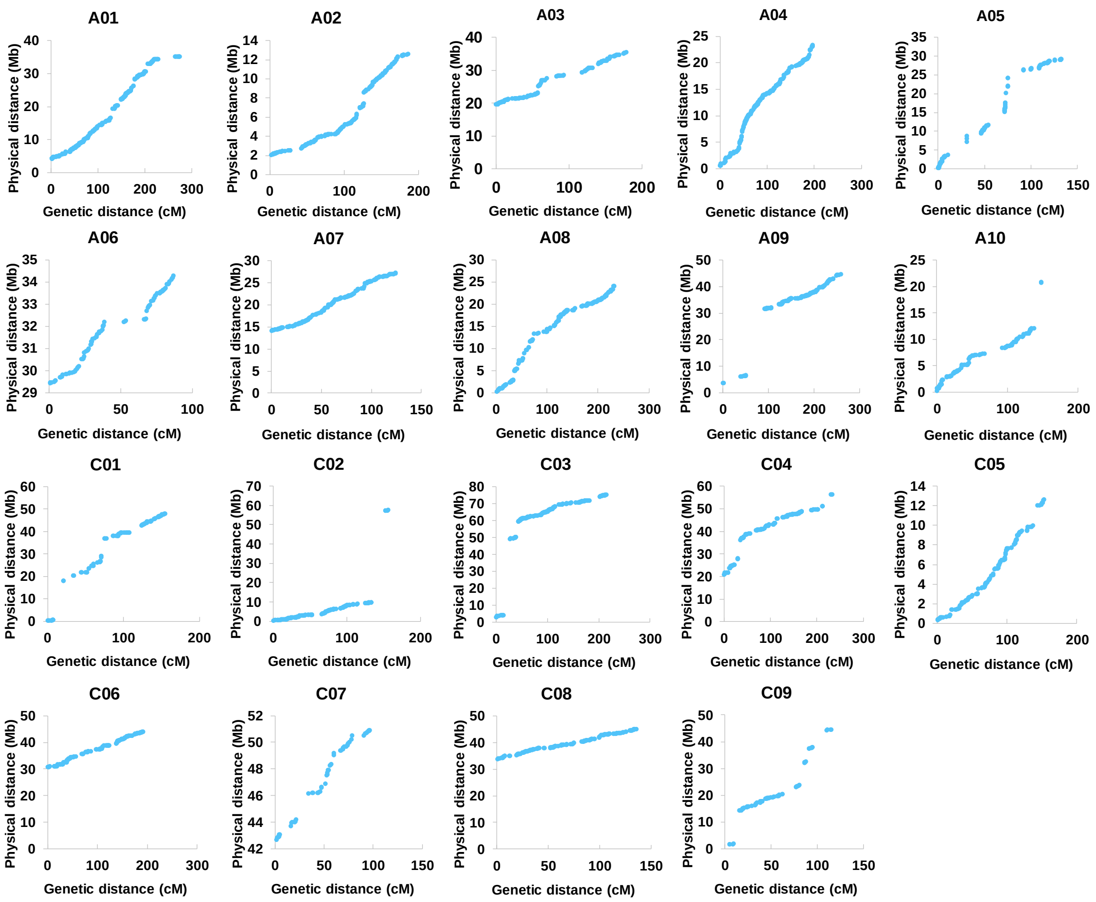

Figure S2. Collinearity analysis of the genetic map and physical map in *B. napus*. The abscissa represents the genetic distance of each linkage group, and the ordinatethe is the physical length of each chromosome, in which the collinearity of the marker in the genome and genetic map is represented in the form of scattered points.

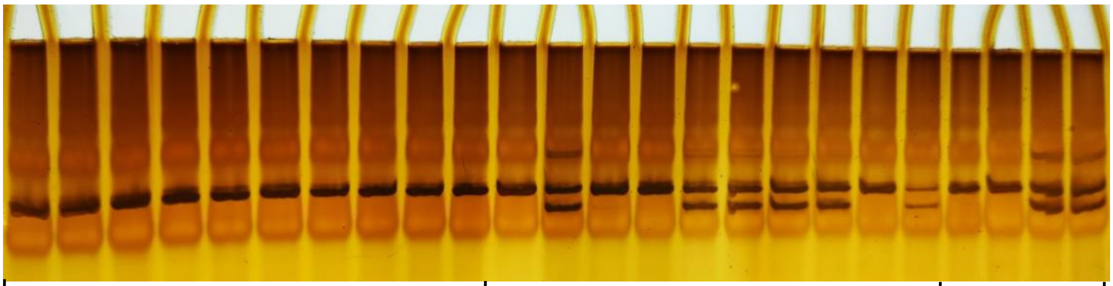

10 extremely early flowering plants | 10 extremely late flowering plants | P1 P1 P2 P2

Figure S3. A small population of 10 extremely early flowering and 10 extremely late flowering plants were analyzed by the cqDTF-C02 locus specific InDel marker C2-5.

Table S8. QTL detection of days to flowering in 158A-SGDH population.

QTL	Chr	Enviroment	CI (cM)	Peak	LOD	PVE (%) [a]	Add [b]
cqDTF-A02	A02	2019-2020 FY-3	153.49-184.07	167.8	3.55	3.83	-0.56
		2020-2021FY-1	147.13-184.07	167.8	4.11	4.24	-0.98
		2020-2021FY-2	164.43-184.07	167.8	2.51	2.77	-0.77
cqDTF-A04	A04	2018-2019FY	97.52-117.8	103.71	2.96	3.86	0.55
		2019-2020 FY-1	137.25-152.19	144.09	4.96	6.27	0.67
		2019-2020 FY-2	137.25-152.19	144.09	5.08	7.38	0.77
		2019-2020 FY-3	137.25-159.75	144.09	4.45	5.16	0.63
		2020-2021FY-1	137.25-152.19	144.09	4.71	5.17	1.04
		2020-2021FY-2	138.25-151.52	144.09	5.6	6.68	1.16
qDTF-A06-1	A06	2020-2021FY-2	0.01-18.97	10.01	4.77	5.51	1.05
qDTF-A06-2	A06	2019-2020 FY-2	34.47-51.91	41.06	3.08	4.43	0.52
cqDTF-A06-3	A06	2018-2019FY	19.38-50.06	29.41	5.27	7.63	0.79
		2019-2020 FY-2	18.97-34.19	29.13	3.49	4.53	0.6
		2020-2021FY-1	24.07-51.06	29.41	5.91	6.54	1.16
cqDTF-A06-4	A06	2019-2020 FY-1	74.88-85.92	85.92	4.88	6.29	0.68
		2019-2020 FY-2	72.1-85.92	79.78	2.74	3.61	0.54
		2019-2020 FY-3	74.88-85.92	85.92	6.7	8.28	0.8
cqDTF-A07-1	A07	2019-2020 FY-1	105.47-119.66	111.54	7.3	9.57	0.82
		2019-2020 FY-2	104.94-117.69	108.16	4.56	6.61	0.71
		2019-2020 FY-3	97.46-124.13	114.91	3.93	4.07	0.55
		2020-2021FY-1	104.69-117.46	108.16	8.23	9.23	1.37
		2020-2021FY-2	105.47-112.66	108.16	6.17	7.33	1.19
cqDTF-A07-2	A07	2019-2020 FY-3	65.94-95.5	73.81	3.53	3.7	0.53
qDTF-A08-1	A08	2019-2020 FY-1	0.01-24.75	2.01	3.37	3.95	0.52
qDTF-A08-2	A08	2019-2020 FY-1	25.64-46.58	35.73	3.37	4.22	0.55
qDTF-A08-3	A08	2019-2020 FY-1	47.86-91.57	70.17	2.87	3.31	0.48
qDTF-A08-4	A08	2019-2020 FY-2	81.48-102.15	90.48	3.16	4.6	0.6

qDTF-A09-1	A09	2019-2020 FY-3	212-230.18	220.15	2.91	3.03	0.47
cqDTF-A09-2	A09	2018-2019FY	231.14-258.24	251.44	3.03	3.93	0.57
		2019-2020 FY-3	230.58-258.24	233.95	2.8	3.02	0.48
qDTF-C01-1	C01	2019-2020 FY-2	0.01-20.89	8.04	2.6	3.65	-0.55
qDTF-C01-2	C01	2018-2019FY	107.26-139.54	130.07	2.96	3.86	0.55
cqDTF-C02	**C02**	**2018-2019FY**	**12.43-33.54**	**23.68**	**8.86**	**13.54**	**1.07**
		**2019-2020 FY-1**	**20.31-41.86**	**34.1**	**10.95**	**15.12**	**1.06**
		**2019-2020 FY-2**	**18.06-33.54**	**33.54**	**10**	**15.67**	**1.12**
		**2019-2020 FY-3**	**19.46-38.48**	**31.56**	**11.85**	**15.32**	**1.1**
		**2020-2021FY-1**	**30.72-51.43**	**40.86**	**22.92**	**32.04**	**2.59**
		**2020-2021FY-2**	**30.72-51.43**	**40.86**	**21.77**	**31.42**	**2.52**
cqDTF-C04-1	C04	2020-2021FY-1	32.59-47.22	46.09	2.7	2.7	1.06
		2020-2021FY-2	33.59-76.33	46.09	2.94	3.17	1.11
cqDTF-C04-2	C04	2020-2021FY-1	69-14-119.1	88.82	3.03	3.1	1.11
		2020-2021FY-2	76.73-118.1	88.82	2.97	3.28	1.12
cqDTF-C05	C05	2019-2020 FY-2	0.01-46.11	31.62	2.85	3.74	0.54
		2019-2020 FY-3	0.01-61.83	37.52	2.59	2.76	0.46
		2020-2021FY-2	0.01-66	31.62	2.53	2.74	0.73
cqDTF-C06	C06	2018-2019FY	38.84-44.18	42.05	7.91	11.79	0.99
		2019-2020 FY-1	31.65-44.86	42.05	9.51	12.64	0.93
		2019-2020 FY-2	3.38-28.99	18.67	8.09	12.46	0.99
		2019-2020 FY-3	39.52-44.18	42.05	12.6	16.13	1.11
		2020-2021FY-1	31.36-45.42	41.37	5.7	6.23	1.12
		2020-2021FY-2	31.36-46.55	41.49	5.31	6.07	1.08
cqDTF-C09	C09	2018-2019FY	75.74-105.07	90.07	4.47	6.45	0.74
		2019-2020 FY-1	63.37-105.98	80.74	3.99	4.54	0.58
		2019-2020 FY-3	75.74-104.07	88.66	4.46	5.13	0.64
		2020-2021FY-1	60.37-107.98	78.74	3.15	3.38	0.86
		2020-2021FY-2	62.37-106.98	88.66	3.04	3.13	0.81

[a] Proportion of the phenotypic variation explained by the QTL; [b] + and − indicate the direction of the additive effect.
